# Mind the gap: Pilot of the FastAsleep digital insomnia intervention in simple English for linguistic minorities in Sweden

**DOI:** 10.1016/j.invent.2026.100973

**Published:** 2026-06-25

**Authors:** Martin Kraepelien, Olof Molander, Moa Pontén, Nitya Jayaram-Lindström, Susanna Jernelöv

**Affiliations:** a Centre for Psychiatry Research, Department of Clinical Neuroscience, Karolinska Institutet & Stockholm Health Care Services, Stockholm, Sweden; b Division of Psychology, Department of Clinical Neuroscience, Karolinska Institutet, Stockholm, Sweden

**Keywords:** Linguistic minorities, Insomnia, Behavioural intervention, Digital intervention, Self-guided

## Abstract

**Background:**

Insomnia is a pervasive global mental health issue, and interventions based on Cognitive Behavioural Therapy for Insomnia (CBT-I) are the recommended first-line treatment. However, barriers such as language skills often prevent linguistic minority populations from accessing CBT-I, exacerbating a significant treatment gap. FastAsleep is a Swedish brief digital self-guided behavioural intervention for insomnia.

**Aims:**

This study aimed to investigate the feasibility of providing FastAsleep in simple English as a proof-of-concept, to enable multilingual access to scalable evidence-based insomnia care for non-Swedish speakers living in Sweden.

**Methods:**

We conducted a single-group pilot study recruiting 11 English-speaking participants nationwide in Sweden via online advertisements. The 4-week, self-guided, sleep-restriction-focused intervention required only brief onboarding and follow-up check-ins via phone with study personnel. Adherence data and self-reported outcomes (e.g., insomnia severity, depression, anxiety) were collected digitally, and all time spent by staff on communication was registered. Trial registered at OSF (https://osf.io/ahvf6).

**Results:**

The participants were born across ten different countries. Adherence to the intervention was high, and staff time averaged only 30 min per participant. Intervention credibility and English material quality were rated as high. Preliminary outcomes showed large reductions in symptoms of insomnia severity (within-group effect size [95% CI] 1.55 [0.59, 2.47]) and reductions in depression similar to FastAsleep in Swedish, but anxiety reductions were seemingly smaller.

**Conclusions:**

The results demonstrate that FastAsleep, as a multilingual approach to digital insomnia care, is feasible and preliminary resource-efficient.

## Highlights

• The Simple-English insomnia tool is feasible for linguistic minorities in Sweden.
• The small sample included participants born across ten different countries.
• Staff time averaged 30 min per participant, indicating efficiency.
• Preliminary results show promising reductions in insomnia severity.
• The approach provides a template for future multilingual sleep care.

## 1 Background

Insomnia disorder is widespread and first line treatment should be Cognitive Behavior Therapy for Insomnia (CBT-I), according to international guidelines (Edinger et al., 2021; Qaseem et al., 2016; Ree et al., 2017; Riemann et al., 2023). FastAsleep (Jernelöv et al., 2023) is a brief self-help intervention based on the central behavioural components from CBT-I: sleep restriction-therapy and stimulus control (Baglioni et al., 2022). The intervention showed promising results in a feasibility study in a Swedish-speaking population (Jernelöv et al., 2023). In a factorial optimization trial, we showed that two central features of FastAsleep, automated reminders and an optimized graphical user interface, were important for high engagement with the intervention (Hentati et al., 2025). A brief self-help intervention such as FastAsleep could be ideally suited as a low-cost initial step within an insomnia stepped-care model (Baglioni et al., 2023). Poor sleep, such as insomnia, is often more common in several minority groups (Hughes et al., 2023). Linguistic minorities may also have problems related to health communication in a second language (Zhao et al., 2021). In Sweden, a significant treatment gap exists for both native English speakers and other linguistic minorities, who may prefer English when seeking healthcare. The aim of this study was to investigate the feasibility for providing the FastAsleep digital intervention in simple English to non-native Swedish speakers in Sweden. Outcomes were used as quality indicators and were benchmarked against the previous feasibility-study of FastAsleep delivered in Swedish (Jernelöv et al., 2023).

## 2 Methods

This study was conducted within the framework of The Mind the Gap-consortium (Molander et al., 2024) (mindthegapstudies.com). The prospective single-group feasibility study was registered on OSF (https://osf.io/v38ub). All procedures involving human subjects/patients were approved by the Swedish Ethical Review Authority (2023-07651-01).

To reach participants, two approaches were used: non-paid dissemination via community outreach and organic social media, followed by paid social media advertisements featuring a short video clip in English. Participant enrolment took place between March 21, 2024, and June 12, 2024. Digital and verbal informed consent was obtained from all participants. The inclusion procedure required participants to register interest, sign informed consent, and complete screening questionnaires on a secure platform. Those not excluded underwent a telephone interview which served as both the final clinical assessment and onboarding session. Inclusion criteria encompassed: age ≥ 18 years; a diagnosis of Insomnia Disorder according to DSM-5; moderate to severe insomnia (Insomnia Severity Index, ISI (Bastien et al., 2001) ≥15); and basic proficiency in reading and writing English. Exclusion criteria included recent changes in antidepressant medications, severe depressive symptoms (Patient Health Questionnaire-9, PHQ-9 (Kroenke et al., 2001) ≥15; see Results for post-hoc deviations), presence of suicidal ideation (PHQ-9 item 9 ≥ 1), prior or concurrent CBT-I, and contraindications or hinders for sleep restriction (e.g., bipolar disorder, narcolepsy, sleep apnea, heart failure, night work, or alcohol/drug abuse).

**Fig. 1:**
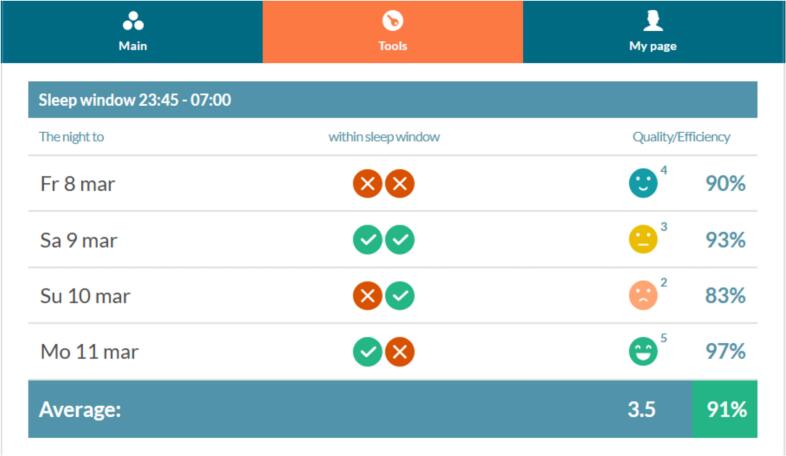
Screenshot of the main component of the intervention, the sleep window-tool.

FastAsleep is a brief, four-week self-guided version of CBT-I primarily focused on sleep restriction therapy delivered via a central Sleep window-tool. Details of the intervention are described elsewhere (Jernelöv et al., 2023). The telephone interviews before and after FastAsleep was seen as an integrated part of the intervention, necessary for high engagement. For this study, author MK (who co-created FastAsleep with SJ) drafted the translation of the intervention content into simple English, to maintain the integrity of the clinical content. To ensure linguistic simplicity and correctness, the resulting translated text was cross-checked using digital tools (Google Translate and Gemini). Generative Large Language Models were not used to generate new texts or other intervention content. Material and vignettes focused on insomnia behavior, requiring no major cultural adaptations. The user-friendly interface (Fig. 1) fosters behavioural engagement compared to more basic user interfaces (Hentati et al., 2025, Hentati et al., 2021).

To address the study's reach, descriptive statistics were employed to characterize the enrolled sample in terms of demographics, country of birth, native languages, and baseline symptom severity scores. Participant adherence and time spent by clinicians were logged in the digital platform. The same adherence measures as in the previous feasibility study (Jernelöv et al., 2023) were used, for benchmarking purposes, including number of daily registrations in the Sleep window-tool (Fig. 1). Intervention credibility was measured during the intervention with a five-item version of the Credibility/Expectancy Questionnaire (CEQ) (Borkovec and Nau, 1972). Utility and satisfaction was assessed quantitatively and qualitatively post intervention with the Internet Evaluation and Utility Questionnaire (IEUQ) (Thorndike et al., 2008). Selected quotes illustrate the range of positive and negative feedback provided in the IEUQ free text-items. The language adaptation was assessed post intervention, using three statements rated from 0 (Strongly Disagree) to 10 (Strongly Agree) on clarity, tone, and cultural sensitivity. Participants were also asked to report any negative effects due to the intervention.

Insomnia severity was measured with ISI (Bastien et al., 2001) up to the 3-month follow-up assessment. Depression symptoms were measured with the PHQ-9 (Kroenke et al., 2001) and anxiety symptoms were measured with the Generalized Anxiety Disorder 7-Item Scale (GAD-7) (Spitzer et al., 2006). Both measures were also administered up to the 3-month follow-up assessment. The symptom measures were used as quality indicators in the benchmark comparison with FastAsleep in Swedish. In addition, functional impairment was measured using the 12-item World Health Organization Disability Assessment Schedule 2.0 (WHODAS) (Üstün et al., 2010) before and after the intervention, standardized total score ranging from no (0) to complete (100) disability. Quantitative outcomes were presented with descriptive statistics and within-group effect-sizes.

## 3 Results

**Fig. 2:**
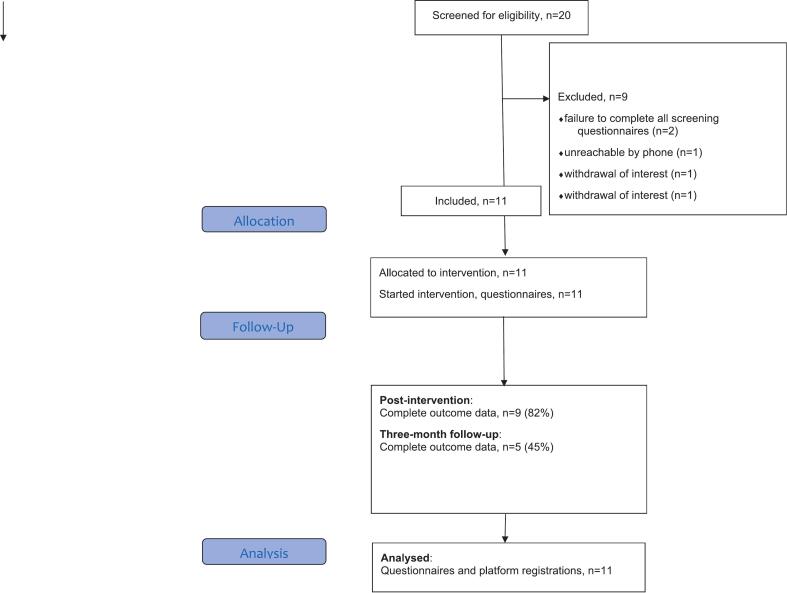
Study flow chart.

The recruitment process (Fig. 2) demonstrated that non-paid outreach was insufficient, yielding only five expressions of interest and no inclusions, whereas the transition to paid social media advertisements proved crucial. Five of the 11 recruited participants met the pre-specified exclusion threshold for depressive symptoms (PHQ-9 ≥ 15). However, based on high self-reported motivation to engage with the sleep intervention and clinical assessment of potential benefit, the research team exercised clinical discretion to include these participants, as the primary focus remained on the insomnia intervention. Participant characteristics, including a diverse range of birth countries and native languages, are summarized in Table 1.

**Table 1:** Participant characteristics (n = 11).

Variable	
Female, n (%)	4 (36%)
Age, mean (range)	46.5 (31–70)
In a relationship, n (%)	7 (64%)
Highest level of education, n (%)	
Primary education	0 (0%)
Secondary education	1 (9%)
College/university	8 (73%)
Other	2 (18%)
Current occupation status, n (%)	
100%	8 (73%)
75%	1 (9%)
50%	0 (0%)
25%	1 (9%)
0%	1 (9%)
Current self-reported financial situation, n (%)	
Very poor	0 (0%)
Poor	1 (9%)
Neither poor or good	2 (18%)
Good	5 (45%)
Very good	3 (27%)
Years in Sweden, mean (range)	11.4 (0–41)
Swedish citizenship, n (%)	7 (64%)
Residence permit, n (%)	4 (36%)
Language skills - Swedish, n (%)	
None or beginner	4 (36%)
Basic or elementary knowledge	1 (9%)
Intermediate knowledge	2 (18%)
Advanced or fluent	4 (36%)
Native speaker	0 (0%)
Language skills - English, n (%)	
None or beginner	0 (0%)
Basic or elementary knowledge	0 (0%)
Intermediate knowledge	0 (0%)
Advanced or fluent	7 (64%)
Native speaker	4 (36%)
Native countries represented	Philippines (n = 2), Bangladesh, Brazil, Cyprus, Germany, Greece, Hong Kong, Iran, Romania, Zimbabwe
Native languages represented	English (n = 3), Portuguese, Greek, Tagalog, Filipino, Persian, Bengali, Romanian, Shona

The average duration of the initial onboarding and inclusion phone call was 22 min, and the follow-up call averaged 8 min, resulting in a total mean staff interaction time of 30 min per participant (SD = 7.6 min). Adherence to the intervention was high and comparable to the previously published Swedish version, with most participants logging their nights in the sleep window-tool daily or almost daily (Table 2).

**Table 2:** Quality indicators of FastAsleep in English, benchmarked against FastAsleep in Swedish (Jernelöv et al., 2023).

Quality indicator	FastAsleep in Swedish, n = 30 (Jernelöv et al., 2023)	FastAsleep in English, n = 11
Adherence, number of registered nights, M (SD) [95% CI]	25.0 out of 28 (5.8) [22.8, 27.2]	21.5 out of 28 (8.4) [15.8, 27.1]
Number of sleep window revisions, M	3.6 out of 6	2.5 out of 6
Bedtime adherence (%)	74%	76%
Rise-time adherence (%)	72%	65%
Treatment credibility, CEQ, M (SD) [95% CI]	33.9 (9.9) [30.1, 37.6]	33.1 (9.9) [27.5, 38.7]
Insomnia severity, ISI (range 0–28), M (SD) [95% CI]		
Screen	18.9 (3.5) [17.6, 20.2]	19.2 (3.4) [16.9, 21.5]
Pre	16.5 (2.9) [15.4, 17.6]	17.5 (3.0) [15.5, 19.5]
Mid	11.0 (4.2) [9.4, 12.6]	14.3 (3.5) [11.8, 16.8]
Post	10.0 (4.2) [8.4, 11.5]	12.7 (4.2) [9.7, 15.7]
Fu3	9.1 (4.9) [7.3, 11.0]	8.4 (3.6) [4.0, 12.8]
Within-group effect size [95% CI] screen to post	2.30 [1.65, 2.95]	1.55 [0.59, 2.47]
Depression symptoms, PHQ-9 (range 0–27), M (SD) [95% CI]		
Screen	8.4 (3.6) [7.1, 9.7]	13.1 (5.4) [9.5, 16.7]
Pre	7.8 (4.1) [6.2, 9.3]	11.7 (6.9) [7.1, 16.3]
Mid	4.4 (3.4) [3.1, 5.7]	9.4 (5.1) [5.8, 13.0]
Post	4.3 (4.6) [2.6, 6.1]	7.6 (5.5) [3.3, 11.8]
Within-group effect size [95% CI] screen to post	0.98 [0.44, 1.52]	0.80 [0.02, 1.54]
Anxiety symptoms, GAD-7 (range 0–21), M (SD) [95% CI]		
Screen	7.0 (3.1) [5.9, 8.1]	10.7 (6.6) [6.3, 15.2]
Pre	4.7 (2.8) [3.7, 5.8]	10.3 (6.3) [6.0, 14.5]
Mid	2.6 (2.3) [1.7, 3.4]	9.3 (5.8) [5.2, 13.4]
Post	2.0 (2.6) [1.0, 3.0]	7.8 (6.8) [2.5, 13.0]
Within-group effect size [95% CI] screen to post	1.59 [1.01, 2.18]	0.33 [−0.35, 1.00]

CEQ, Credibility/Expectancy Questionnaire; ISI, Insomnia Severity Index; PHQ-9, Patient Health Questionnaire-9; GAD-7, Generalized Anxiety Disorder 7-Item Scale.

The intervention evaluation with IEUQ is presented in Supplementary Table A1. All participants consistently provided high ratings regarding the ease of understanding of the information. Three non-serious adverse events (increased daytime sleepiness) were reported by two participants.

Participant ratings regarding the language adaptation (*n* = 9) were high across all three domains (0–10 scale). The perception of ease of understanding averaged 9.0 (SD = 1.3), while the appropriateness of tone and style scored 9.1 (SD = 0.8). Ratings for the language's ability to avoid stereotypes and consider cultural diversity were 8.9 (SD = 1.6).

Baseline insomnia severity (ISI) scores were comparable to those reported in the Swedish FastAsleep version, with large reductions on ISI (insomnia severity) observed post-intervention, although with a numerically smaller mean within-group effect size compared to the benchmark study (Jernelöv et al., 2023). Baseline scores for PHQ-9 and GAD-7 (anxiety) were noticeably higher in the English sample, with similarly sized reductions on PHQ-9 (depression), but reductions in anxiety symptoms following the intervention being smaller (Table 2) compared to benchmark. Regarding functional impairment, the sample reported a mean baseline WHODAS-12 score of 30.1 (SD = 19.4, 95% CI = 17.1, 43.2), and post-intervention, this score was 16.9 (SD = 17.6, 95% CI = 3.4, 30.4), indicating a reduction in self-reported functional disability, within-group effect size of *d* = 0.89 (95% CI = 0.09, 1.65).

## 4 Discussion

The aims of this study were to investigate the feasibility of adapting the FastAsleep digital intervention into simple English for linguistic minorities in Sweden and to assess key process indicators for future multilingual scale-up. The study demonstrates that adapting FastAsleep into simple English is a viable template for further language adaptations, though major language groups like Arabic would require dedicated linguistic expertise.

The enrolled sample presented with several unique characteristics including a high proportion of male participants compared to the previous study (Jernelöv et al., 2023). The sample demonstrated a desired breadth of participant background, featuring diverse countries of birth and native languages, a finding that contrasts with a previous PTSD-study within the Mind the Gap-initiative, which drew heavily from larger Anglophone countries (Molander et al., 2025). Clinically, the sample reported higher baseline depression scores than the Swedish FastAsleep cohort, yet descriptively matched the Swedish cohort with comparable post-intervention reductions in depression (though scores may not be strictly comparable) and large reductions in insomnia. In contrast, any reduction in co-occurring anxiety symptoms appeared smaller than in the Swedish study.

Some limitations of this study must be considered. The findings suggest that while this simple-English adaptation successfully bridged a treatment gap for a diverse, multi-country sample, the high education and proficiency levels limit generalizability. Simple English may not suffice for lower-proficiency minorities, who likely require full translations and tailored outreach. The small sample size and the absence of a randomized control group mean that no definitive conclusions regarding efficacy can be drawn. The reliance on recruitment by digital ads likely biased the sample towards individuals who are digitally adept social media users. Despite these limitations, this pilot marks a crucial step in providing evidence-based care to populations previously excluded from interventions. These findings pave the way for a multilingual rollout of FastAsleep in Sweden, though larger studies in multiple minority languages are warranted.

## CRediT authorship contribution statement

The study and its research questions were designed and conceptualized by all authors. The data was collected and analyzed by MK. The manuscript was drafted by MK. All authors reviewed and commented on subsequent versions of the manuscript, and read and approved the final manuscript.

## Transparency declaration

The lead author affirms that the manuscript is an honest, accurate, and transparent account of the study being reported; that no important aspects of the study have been omitted; and that any discrepancies from the study as planned have been explained.

## Funding

This study was supported by the Swedish 10.13039/501100005348Ministry of Health and Social Affairs (MK, SJ, grant number S2018/03855/FS), Region Stockholm (all authors), 10.13039/501100009750Kamprad Family Foundation (NJ-L, grant number 20243396) and KI Funds - Rut and Arvid Wolff Memorial Foundation (MK, grant number 2023-02493). The funders had no influence on study design, data collection, analysis or publication.

## Declaration of competing interest

The authors declare that they have no known competing financial interests or personal relationships that could have appeared to influence the work reported in this paper.

## Data Availability

The data are available from the corresponding author, MK, upon reasonable request.

## Appendix A Supplementary data

Supplementary Table A1 Internet Evaluation and Utility Questionnaire (IEUQ), quantitative questions (1–13), post intervention (*n* = 9).
