# Supplementary material for: Mind the gap: Pilot of the FastAsleep digital insomnia intervention in simple English for linguistic minorities in Sweden
Source: Internet Interv. 2026 Jun 25;45:100973. doi: 10.1016/j.invent.2026.100973 (PMC13333308; doi:10.1016/j.invent.2026.100973)
Supplement: Supplementary Table A1 — Internet Evaluation and Utility Questionnaire (IEUQ), quantitative questions (1–13), post intervention (n = 9). [file mmc1.docx]

**Supplementary table A1.** Internet Evaluation and Utility Questionnaire (IEUQ), quantitative questions (1-13), post intervention (n=9).

| Question | 0. Not at all | 1. Slightly | 2. Somewhat | 3. Mostly | 4. Very | 5. NA |
| --- | --- | --- | --- | --- | --- | --- |
| **1. How easy was the web program to use?** | 0 | 1 | 3 | 2 | 3 | 0 |
| **2. How convenient was the web program to use?** | 0 | 3 | 1 | 2 | 3 | 0 |
| **3. How much did the web program keep your interest and attention?** | 0 | 2 | 3 | 2 | 1 | 0 |
| **4. How much did you like the web program?** | 0 | 2 | 3 | 2 | 1 | 0 |
| **5. How much did you like the way the web program looked?** | 0 | 2 | 4 | 2 | 1 | 0 |
| **6. How worried were you about your privacy in using this web program?** | 6 | 1 | 2 | 0 | 0 | 0 |
| **7. How satisfied were you with the web program?** | 0 | 1 | 3 | 4 | 1 | 0 |
| **8. How good of a fit was the web program for you?** | 1 | 0 | 3 | 5 | 0 | 0 |
| **9. How useful did you find the information in the web program?** | 0 | 1 | 4 | 4 | 0 | 0 |
| **10. How easy was the information to understand?** | 0 | 0 | 0 | 4 | 5 | 0 |
| **11. How much did you feel you could trust the information?** | 0 | 0 | 1 | 2 | 6 | 0 |
| **12. If difficulties continue or return, how likely would you be to come back to this web program?** | 0 | 2 | 4 | 2 | 1 | 0 |
| **13. How good of a method was the Internet for delivering this intervention?** | 0 | 3 | 1 | 2 | 3 | 0 |

Two qualitative quotes from items 14 and 15 to illustrate the variety of experiences reported by participants were “I now go to bed an hour later and am able to go back to sleep when I wake in the night. Without the program I would not have made this change. My husband also has made the change.” (quote illustrating an experience of the program being helpful) and “[I] could not pose questions and get immediate response.” (quote illustrating an experience of the program not being helpful).
